# POTENTIAL RISK OF BRAIN DAMAGE AND POOR DEVELOPMENTAL OUTCOMES IN CHILDREN PRENATALLY EXPOSED TO SARS-COV-2: A SYSTEMATIC REVIEW

**DOI:** 10.1590/1984-0462/2022/40/2020415

**Published:** 2021-05-26

**Authors:** Marcio Leyser, Fernanda Jordão Pinto Marques, Osvaldo José Moreira do Nascimento

**Affiliations:** a University of Iowa, Iowa City, Iowa, United States.; b Universidade Federal Fluminense, Niterói, RJ, Brazil.

**Keywords:** SARS-CoV-2, SARS-CoV, MERS-CoV, Pregnancy, Brain injuries, Coronavirus, SARS-CoV-2, SARS-CoV, MERS-CoV, Gravidez, Lesão cerebral, Coronavírus

## Abstract

**Objective:**

To perform a systematic literature review to analyze existing data on the neurological effects of coronavirus on newborns.

**Data:**

**sources:** We followed the guidelines of the Preferred Reporting Items for Systematic Reviews and Meta-Analyses (PRISMA) and the Preferred Reporting Items for Systematic Review and Meta-Analysis Protocols (PRISMA-P), and searched the PubMed and Embase platforms for the keywords [brain damage OR pregnancy OR developmental outcomes] and [coronavirus OR SARS-CoV-2 OR SARS-CoV OR MERS-CoV] between January 1, 2000 and June 1, 2020.

**Data:**

**synthesis:** Twenty-three reports described the course of pregnant women exposed to SARS-CoV-2, SARS-CoV, or MERS-CoV during the gestational period, eight to SARS-CoV-2, eight to SARS-CoV, and seven to MERS-CoV. No data were found on abnormalities in brain development or on a direct link between the virus and neurological abnormalities in the human embryo, fetus, or children. Spontaneous miscarriage, stillbirth, and termination of pregnancy were some complications connected with SARS/MERS-CoV infection. SARS-CoV-2 is not currently associated with complications in the gestational period.

**Conclusions:**

The literature has no data associating exposure to coronavirus during pregnancy with brain malformations and neurodevelopmental disorders. However, despite the lack of reports, monitoring the development of children exposed to SARS-CoV-2 is essential given the risk of complications in pregnant women and the potential neuroinvasive and neurotropic properties found in previous strains.

## RESUMO

**Objetivo:**

Realizar uma revisão sistemática da literatura para analisar os dados existentes sobre os efeitos neurológicos do coronavírus em recém-nascidos.

**Fontes:**

**de dados:** Esta revisão seguiu as diretrizes dos Principais Itens para Relatar Revisões Sistemáticas e Meta-Análises (*Preferred Reported Items for Systematic Review* - PRISMA) e dos Protocolos dos Principais Itens para Relatar Revisões Sistemáticas e Meta-Análises (*Preferred Reporting Items for Systematic Review and Meta-Analysis Protocols* - PRISMA-P), pesquisando as plataformas PubMed e Embase pelas palavras-chave [*brain damage* (dano cerebral) OU *pregnancy* (gravidez) OU *developmental outcomes* (alterações de desenvolvimento)] e [*coronavirus* (coronavírus) OU SARS-CoV-2 OU SARS-CoV OU MERS-CoV] entre 1º de janeiro de 2000 e 1º de junho de 2020.

**Síntese:**

**dos dados:** Vinte e três relatos descreveram a evolução de mulheres grávidas expostas ao SARS-CoV-2, SARS-CoV ou MERS-CoV durante o período gestacional, oito ao SARS-CoV-2, oito ao SARS-CoV e sete ao MERS-CoV. Não foram encontrados dados sobre anormalidades no desenvolvimento cerebral ou sobre uma ligação direta entre o vírus e alterações neurológicas no embrião, feto ou crianças. Abortamento espontâneo, morte fetal e interrupção da gravidez foram algumas das complicações relacionadas à infecção por SARS/MERS-CoV. Até o momento, o SARS-CoV-2 não está associado a complicações no período gestacional.

**Conclusões:**

Não há dados na literatura que associem a exposição ao coronavírus durante a gravidez com malformações cerebrais e distúrbios do neurodesenvolvimento. No entanto, apesar da falta de relatos, o monitoramento do desenvolvimento de crianças expostas ao SARS-CoV-2 é essencial devido ao risco de complicações em gestantes e às potenciais propriedades neuroinvasivas e neurotrópicas encontradas em cepas anteriores.

## INTRODUCTION

With the emergence of a novel coronavirus (SARS-CoV-2) strain from Wuhan^1^ that has alarmed the international scientific community, lessons learned from the unprecedented Zika (ZIKV)^2^ epidemic inevitably should prompt researchers to raise the following question: *Are babies born to SARS-CoV-2 prenatally infected mothers at risk for stillbirths, prematurity, brain damage, and associated poor developmental outcomes?*

Until the arrival of SARS-CoV-2, only six known strains of coronavirus (CoV) were capable of infecting humans, including those that caused the severe acute respiratory syndrome (SARS-CoV), first outbreak in 2002, and the Middle East respiratory syndrome (MERS-CoV), first outbreak in 2012.^3^ When this article was submitted, according to the World Health Organization (WHO) last Situation report released on October 18, 2020, approximately 39,596,858 cases were confirmed globally, particularly in America.^4^ ^,^ ^5^

CoVs are known to induce both respiratory and extrapulmonary symptoms in humans.^1^ SARS-CoV-2 has a single-stranded positive-sense RNA that belongs to the subfamily *Coronavirinae*, family *Coronaviridae*, order *Nidovirales*. Among the four major genera (*Alphacoronavirus*, *Betacoronavirus*, *Gammacoronavirus,* and *Deltacoronavirus*), sequence analysis revealed that SARS-CoV-2 relates to the *Betacoronavirus* cluster*.* ^6^ Similar to SARS-CoV and MERS-CoV, the SARS-CoV-2 genome encodes nonstructural proteins (such as 3-chymotrypsin-like protease, papain-like protease, helicase, and RNA-dependent RNA polymerase), structural proteins (such as spike glycoprotein), and accessory proteins.^7^

The spectrum of the coronavirus disease 2019 (COVID-19) ranges from asymptomatic infection to mild-to-severe pneumonia that leads to respiratory failure, septic shock, multiple organ failure, and death. Most cases present with fever (98%), fever with cough (83%), and difficulty in breathing (72%).^3^

As our knowledge of this CoV strain is still incipient, we do not have a definitive answer to our query yet. However, we can at least provoke researchers by inviting them to raise additional key questions that could preemptively lead to directives in how the scientific community should tailor the next investigative steps. We propose some ideas for innovative research on the SARS-CoV-2 teratogenic risk, including affinities to congenital neurotropic virus diseases, as seen with ZIKV infection.^2^ The following questions may help guide researchers as they design future studies on this subject matter:

- Is SARS-CoV-2 capable of crossing the placental barrier and induce maternal-fetal transmission?
- Are mothers infected with SARS-CoV-2 at a higher risk of gestational complications that can lead to miscarriages, prematurity, and even secondary neurological and developmental problems in their offspring compared to other respiratory viruses?
- Is SARS-CoV-2 a strongly neurotropic virus that could either immediately (by direct viral activity) or indirectly (by the host’s immune responses) cause fetal brain damage in the early stages of pregnancy or change fetal brain networks in later stages of pregnancy, consequently affecting developmental outcomes?

While specific studies on the interplay between SARS-CoV-2 and fetal growth and development, including viral interference in the development and maturation of the fetal brain, are still pending, we suggest that the best way to respond to the aforementioned questions may be through both a systematic and a literature review. In this paper, we analyzed existing data based on the current pandemic and previous coronavirus epidemics affecting pregnant women. Additionally, while other viruses - such as ZIKV and cytomegalovirus (CMV) - stem from different taxonomic classifications and phenotypic characteristics, the fact that they can induce severe brain injuries during the fetal embryonic life also justifies an analogy with SARS-CoV-2 from a pathophysiological standpoint. Below, we provide examples organized around the questions above.

## METHOD

**Figure 1:**
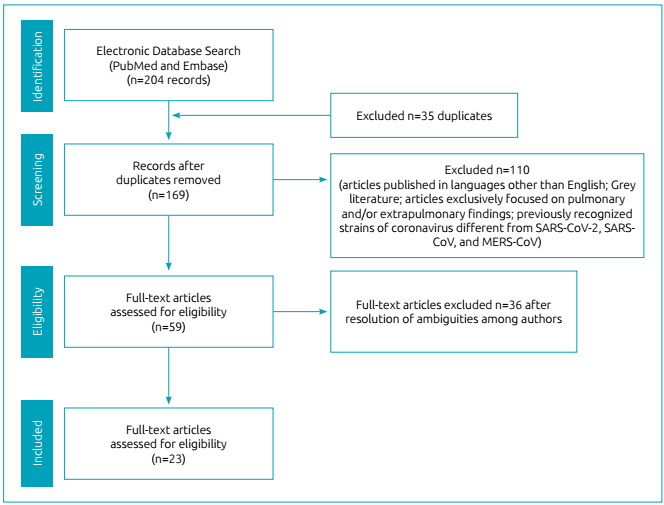
Preferred Reported Items for Systematic Review Flow Diagram.

Guidelines from the Preferred Reporting Items for Systematic Reviews and Meta-Analyses (PRISMA) and the Preferred Reporting Items for Systematic Review and Meta-Analysis Protocols (PRISMA-P) were applied to conduct the systematic literature search (Figure 1). We searched the PubMed and Embase databases for abstracts and full texts of clinical trials, meta-analyses, randomized controlled trials, systematic reviews, and case reports containing the keywords [brain damage OR pregnancy OR developmental outcomes] and [coronavirus OR SARS-CoV-2 OR SARS-CoV OR MERS-CoV], published between January 1, 2000 and June 1, 2020.

Articles produced in languages other than English and Grey literature were not considered for further evaluation. We screened the titles/abstracts to identify articles of interest. Subsequently, we assessed the original array of papers for inclusion. Discussions among the reviewers resolved any remaining disagreements or ambiguities.

We also conducted a random search in PubMed and Embase for recent reviews, reports, and letters regarding both SARS-CoV-2 and ZIKV biological properties to assess further and compare whether potential increased neurotropism risks for SARS-CoV-2 could contribute to brain damage and poor developmental outcomes similar to what was observed with ZIKV.^2^

Inclusion criteria were studies reporting on pregnant women who contracted SARS-CoV-2, SARS-CoV, or MERS-CoV; or brain development or brain damage or direct virus-mediated neurological abnormalities in the human embryo, fetus, or children caused by SARS-CoV-2, SARS-CoV, or MERS-CoV.

Exclusion criteria were articles published in languages other than English; Grey literature; articles exclusively focused on pulmonary and/or extrapulmonary findings; previously recognized coronavirus strains different from SARS-CoV-2, SARS-CoV, or MERS-CoV.

## RESULTS

Corresponding to the inclusion criteria established for this systematic review, we found eight articles^8^ ^,^ ^9^ ^,^ ^10^ ^,^ ^11^ ^,^ ^12^ ^,^ ^13^ ^,^ ^14^ ^,^ ^15^ describing the course of pregnant women who contracted SARS-CoV during the gestational period. Seven articles^16^ ^,^ ^17^ ^,^ ^18^ ^,^ ^19^ ^,^ ^20^ ^,^ ^21^ ^,^ ^22^ reported on pregnant women exposed to MERS-CoV, and eight articles^23^ ^,^ ^24^ ^,^ ^25^ ^,^ ^26^ ^,^ ^27^ ^,^ ^28^ ^,^ ^29^ ^,^ ^30^ related COVID-19 to the gestational period. We were unable to retrieve any articles on related abnormalities in the embryonic or fetal stages of brain development or reporting a direct link between the virus and neurological abnormalities in the human embryo, fetus, or children induced by SARS-CoV, MERS-CoV, or SARS-CoV-2. These studies are summarized in Tables 1, 2, and 3.

**Table 1:** Characteristics of studies related to SARS-CoV.

SARS-CoV	Author/year	Study population	Study design and intervention	Outcome measures	Conclusions
	Ng et al., 2004^8^	n=12 pregnant women infected with SARS-CoV.	Review	Four pregnant women died; two had a spontaneous miscarriage and termination of pregnancy; no newborn had SARS-CoV infection.	Coronavirus is unlikely to be transmitted via the intrauterine route.
	Lau et al., 2004^9^	n=1 32-year-old woman.	Case report	Incidence of generalized seizures with a positive RT-PCR for SARS-CoV in the cerebrospinal fluid.	SARS-CoV can infect multiple organ systems, and CNS can possibly be affected.
	Yudin et al., 2005^10^	n=1 33-year-old pregnant woman.	Case report	Labor occurred spontaneously at term, and a healthy female newborn was delivered.	No evidence of perinatal transmission.
	Stockman et al., 2004^11^	n=2 pregnant SARS case-patients.	Letter	Neither of the two pregnant women had severe negative outcomes.	Few cases have been studied to define clearly the risks of pregnant women infected with SARS-CoV.
	Robertson et al., 2004^12^	n=1 36-year-old pregnant woman.	Case report	Healthy female infant was delivered without complications.	Long-term follow-up of infants is needed to characterize the pregnancy-related risks.
	Lam et al., 2004^13^	n=10 pregnant and 40 non-pregnant female patients.	Case-control	Four required endotracheal intubations, and six were admitted to the intensive care unit (ICU). Three deaths in the pregnant group.	Pregnant women with SARS experience a worse clinical course and poorer outcomes compared with non-pregnant women.
	Shek et al., 2003^14^	n=5 liveborn infants.	Case series	No manifestations or radiological, hematologic, or biochemical evidence suggestive of SARS.	Newborns of mothers with SARS did not develop the disease.
	Li et al., 2005^15^	n=12 pregnant women infected with SARS-CoV included in this review	Review	No evidence suggests that the virus is transmitted transplacentally.	Vertical transmission of SARS-CoV from infected mothers to their newborns has not been observed.

**Table 2:** Characteristics of studies related to MERS-CoV.

MERS-CoV	Author/year	Study population	Study design and intervention	Outcome measures	Conclusions
	Assiri et al., 2016^16^	n=5 pregnant women.	Case report	Two patients died, two cases resulted in perinatal death, one pregnancy led to intrauterine fetal demise, and one infant died after an emergency cesarean section.	MERS-CoV may pose serious health risks to both mothers and infants during pregnancy.
	Alfaraj et al., 2019^17^	n=2 pregnant women.	Case report	Both patients did not deliver during hospitalization and subsequently delivered a healthy infant at term.	The outcome was favorable in most pregnancies associated with MERS-CoV cases.
	Jeong et al., 2017^18^	n=1 39-year-old pregnant woman.	Case report	Benign maternal course that resulted in full recovery with subsequent healthy full-term delivery.	Further studies with a larger sample size will expand the knowledge of pathophysiology and perinatal outcome.
	Alserehi et al., 2016^19^	n=1 33-year-old pregnant woman.	Case report	Preterm delivery of a male infant without complications.	The pregnancy had a favorable outcome for the newborn.
	Malik et al., 2016^20^	n=1 32-year-old woman.	Case report	Preterm delivery of a male infant without complications. The mother presented a progressive worsening of clinical status, leading to death.	MERS-CoV infection and pregnancy were a fatal combination in this case.
	Park et al., 2016^21^	n=1 39-year-old woman.	Case report	Placental abruption; urgent C-section.	The pregnancy had a favorable outcome for both mother and newborn.
	Payne, 2014^22^	n=1 39-year-old pregnant woman.	Case report	Second-trimester stillbirth in a pregnant woman with MERS-CoV infection.	MERS-CoV infection during pregnancy may pose serious health risks to both mother and fetus.

**Table 3:** Characteristics of studies related to SARS-CoV-2.

SARS-CoV-2	Author/year	Study population	Study design and intervention	Outcome measures	Conclusions
	Juan et al., 2020^23^	n=266 pregnant women.	Systematic review	One case of neonatal asphyxia and one of neonatal death. The case reports described two maternal deaths among pregnant women with COVID-19.	Clinical characteristics of pregnant women with COVID-19 are similar to those of non-pregnant adults with COVID-19.
	Egloff et al., 2020^24^	n=179 newbornstested for SARS-CoV-2 at birth.	Review	Transmission was suspected in eight cases, five with positive nasopharyngeal SARS-CoV-2 RT-PCR and three with SARS-CoV-2 IgM.	Current data indicate very rare maternal-fetal transmission but are largely incomplete.
	Lamouroux et al., 2020^25^Alzamora et al., 2020^26^	n=68 deliveries and 71 neonates with maternal infection.n=1 41-year-old pregnant woman with COVID-19.	Case report	Neonatal infection was diagnosed within 48 hours of life in four cases.Neonatal nasopharyngeal swab was positive for SARS-CoV-2.	More definitive evidence is needed for counseling pregnant women on the risk of congenital infection.Vertical transmission remains controversial.
	Karimi-Zarchi et al., 2020^27^	n=31 infected pregnant mothers.	Review	No COVID-19 infection was detected in their neonates or placentas. Two mothers died from COVID-19-related respiratory complications after delivery.	There is no evidence of intrauterine transmission. Mothers may be at increased risk for severe respiratory complications.
	Schwartz et al., 2020^28^	n=38 pregnant women.	Review	No deaths among the 38 pregnant women. No confirmed cases of intrauterine transmission of SARS-CoV-2.	There is no evidence of intrauterine or transplacental transmission.
	Chen et al., 2020^29^	n=9 pregnant women.	Retrospective review	No patient developed severe COVID-19 pneumonia or died. No neonatal asphyxia was observed in the newborns.	The clinical characteristics of pneumonia in pregnant women were similar to those reported for non-pregnant adult patients.
	Lu et al., 2020^30^	n=1 22-year-old pregnant woman.	Case report	COVID-19 nucleic acid test was three times negative in the newborn.	There might be no intrauterine infection caused by vertical transmission.

## DISCUSSION

### Is SARS-CoV-2 capable of inducing placental damage?

Data on MERS-CoV or SARS-CoV during pregnancy - and their possible resulting negative impact in terms of stillbirth, premature birth, or on fetal brain development and maturation due to placental insufficiency - remains scant.^15^ ^,^ ^18^ Like SARS and MERS, no cases of intrauterine transmission of SARS-CoV-2 from mothers with COVID-19 to their fetuses have been confirmed. With respect to SARS-CoV-2, a recent systematic review published by Juan et al.^23^ including case series and case reports assessing 266 pregnant women revealed that vertical transmission of SARS-CoV-2 remains controversial, so more data are needed to conclude on this point.

Shanes et al.^31^ recently reported on the placental pathology of 16 patients infected with SARS-CoV-2 and identified no pathognomonic features. However, a higher incidence of chorangiosis was documented. This finding is supported by the pro-inflammatory properties of SARS-CoV-2, which are also responsible for multisystemic complications.^32^ Although larger case series are necessary for further analysis of placental damage, antenatal surveillance for women diagnosed with SARS-CoV-2 is warranted due to the risk of pregnancy and adverse perinatal outcomes.

### Are mothers infected with SARS-CoV-2 at a higher risk of gestational complications that can lead to miscarriages, prematurity, and even secondary neurological and developmental problems in their offspring compared to other respiratory viruses?

Coronavirus infections in pregnant women caused by SARS-CoV and MERS-CoV are associated with gestational complications, especially in mothers with cases of severe illness, and significant death rates.^28^ Among 12 pregnant women who developed SARS during the epidemic, 3 died during pregnancy (25%). MERS-CoV infection has been reported in 11 pregnant women, among whom 10 (91%) presented a direct association with a variety of adverse clinical outcomes.

Conversely, COVID-19 has not led to maternal deaths or significant clinical complications to mothers thus far. Currently, the literature only describes data from pregnant women at the third trimester of pregnancy; larger cohorts will be needed for further analysis.^23^

The cytokine status and hyperinflammation found in pregnant women infected with SARS-CoV-2 may, in theory, increase the risk for neurodevelopmental disorders in neonates.^33^ Indeed, as a direct correlation between the virus and host immune response mechanisms of action and the damage to developing neural progenitor cells of the human embryo or fetus in the early stages of pregnancy cannot be proven yet, severe maternal hypoxia induced by SARS-CoV-2 should theoretically be considered a potential risk factor for prematurity, stillbirth, or fetal brain damage.

### Is SARS-CoV-2 a strongly neurotropic virus that could either immediately (by direct viral activity) or indirectly (by the host’s immune responses) cause fetal brain damage in the early stages of pregnancy or change fetal brain networks in later stages of pregnancy, consequently affecting developmental outcomes?

Desforges et al.^34^ revealed that human respiratory coronaviruses are naturally neuroinvasive and neurotropic. The authors state that the potential neuropathological consequences in genetically or otherwise susceptible individuals might occur regardless of the presence of additional environmental insults.

Although less common than respiratory symptoms, central nervous system (CNS) involvement is possible and has been described.^35^ The pathophysiology of CNS involvement induced by MERS-CoV includes dipeptidyl peptidase-4 (DPP-4) as the receptor for cell binding and access.^3^ MERS-CoV might gain access to the human CNS by disrupting the blood-brain barrier via lymphatic vessels or by alternative routes, where cells expressing DPP-4 could support virus replication.^36^ SARS-CoV is known to be responsible for neurological complications due to the direct viral infection of neuronal cells in the cortex and hypothalamus.^9^ Nevertheless, the pathogenesis of previous neurological involvement due to SARS-CoV is unclear. Some candidates such as chemokine Mig might be involved in the brain immunopathology by attracting immune effector cells to the site of virus infection.^37^

SARS-CoV-2 uses angiotensin-converting enzyme 2 (ACE2) as its receptor, contributing to human cell infection. The RNA expression profile of ACE2 in the trophoblast seems to be low between 6 and 14 weeks; therefore, mother-to-fetus transmission of SARS-CoV-2 during the first trimester is unlikely.^28^ Because the ACE2 receptor is displayed in many biological tissues, including the brain, it might function as part of a biological mechanism that could lead to neurological complications.^38^ Wan et al. analyzed neural cell lines of human and rat specimens that expressed undetectable levels of ACE2 caused by the replication of SARS-CoV within the cells. This finding might explain some viral-related neurological complications.^39^

### SARS-CoV-2, ZIKV, their biological properties, and comparative risks for neurotropism

Viral intrauterine transmission is one of the most serious gestational complications. For example, it can occur in other congenital infectious diseases, such as rubella, cytomegalovirus, herpes, Zika virus, and Ebola virus.^28^

The transmission of SARS-CoV and MERS-CoV has not been documented to occur during pregnancy, although infections caused by these coronaviruses have resulted in severe maternal pneumonia, maternal deaths, and early pregnancy losses.

Interestingly, for over 50 years, ZIKV was an agent responsible for a mild disease in humans, with a few clusters in Asia and Africa. Now, after the recent epidemics, it is recognized as an agent responsible for neurological complications, notably in infants exposed to the virus during the embryonic period. Also, infants exposed to ZIKV during the gestational period are at risk for developmental disabilities due to its intense neurotropism.^2^

ZIKV comprises an RNA genome surrounded by a lipid-rich membrane within an icosahedron-shaped protein structure. This genome is organized in a single strand of RNA that encodes three structural proteins (C, prM/M, and E) and seven nonstructural ones (NS1, NS2A, NS2B, NS3, NS4A, NS4B, and NS5). The largest protein is NS5, responsible for viral RNA encapsulation and viral RNA synthesis. In addition, NS5 proteins have proven to inhibit interferon type I (IFN) signaling to bypass the host’s antiviral defense^40^ and, consequently, neutralize immune responses to the virus.

Sequence analysis revealed that SARS-CoV-2 has the common genome structure of CoVs and belongs to the cluster of *betacoronavirus*. It consists of a single-stranded positive-sense RNA and uses the genomic RNA to encode nonstructural proteins (nsps) to form a replication-transcription complex (RTC) in double-membrane vesicles (DMVs). Homotrimers of S proteins make up the spike on the surface of virus particles, and this is the main mechanism for the viral attachment to the host receptor. The M protein has three transmembrane domains, and it shapes the virions. It requires the E protein for pathogenesis. Also, the N protein is an interferon antagonist and viral-encoded suppressor (VSR) of RNA interference (RNAi), which benefits viral replication.

Based on SARS-CoV-2 biological properties, the virus might theoretically neutralize the maternal immune response by affecting IFN expression, similar to ZIKV. This notion needs to be better explained. As both CoV and ZIKV have pathophysiological mechanisms that interfere in interferon expression, virus spread could be viable, indicating a risk factor for CoV neurotropism and, as a result, risk of damage to progenitor cells of the brain.

### Study limitations

We acknowledge several study limitations in this paper. By far, the most significant aspect is that even after two significant coronavirus epidemics, including two remarkable outbreaks of respiratory diseases,^14^ ^,^ ^15^ the literature is basically supported by case reports or case series, which diminishes the credibility in terms of the quality of evidence. We could not find any cross-sectional, observational, cohort studies of children whose mothers have been affected by SARS-CoV-2, SARS-CoV, and/or MERS-CoV. Moreover, the systematic review search strategy was restricted to only two biomedical literature database sources. While a wider search involving more sources could have provided a larger array of articles, based on the sample gathered by the two aforementioned database platforms, we concluded that, although a few more case reports might have been located, including the addition of languages other than English, distinctions in the quality of study designs would not have been significantly greater at this point.

Even though SARS-CoV-2 somewhat biologically resembles the previous SARS-CoV and MERS-CoV, and data are scant and inconclusive regarding the consistency of both their primary and secondary potential negative outcomes in children prenatally exposed to SARS-CoV-2, we strongly recommend monitoring and surveilling the development of these children during the epidemic cycle. Recommendations for extensive systematic neuroimaging evaluations and referrals to in-depth developmental assessments still need to be further elucidated. At this stage, they should continue to be carried out on a case-by-case basis in term infants exposed to SARS-CoV-2 according to abnormal neurological signs and physical-neurological examinations. As for premature babies, the application of established protocols for this population should continue to govern the extent of both neurological and developmental investigations.

Also, attempts should be made to explain better its viral diversity and ecological drivers for future surveillance and management of emerging zoonoses. As previously proposed for the Zika problematics, perhaps a multilevel research framework^41^ approach would further help to assist researchers when examining the multifaceted gestalt that constitutes this novel virus and better educate us all as we deal with these and many other complex and emergent related challenges.
